# Supplementary material for: Differential cell-intrinsic regulations of germinal center B and T cells by miR-146a and miR-146b
Source: Nat Commun. 2018 Jul 16;9:2757. doi: 10.1038/s41467-018-05196-3 (PMC6048122; doi:10.1038/s41467-018-05196-3)
Supplement: Supplementary file 1 — Supplementary Information [file 41467_2018_5196_MOESM1_ESM.pdf]

Supplementary Figures

Differential cell-intrinsic regulations of germinal center B and T cells by miR-146a and miR-146b

Cho et al

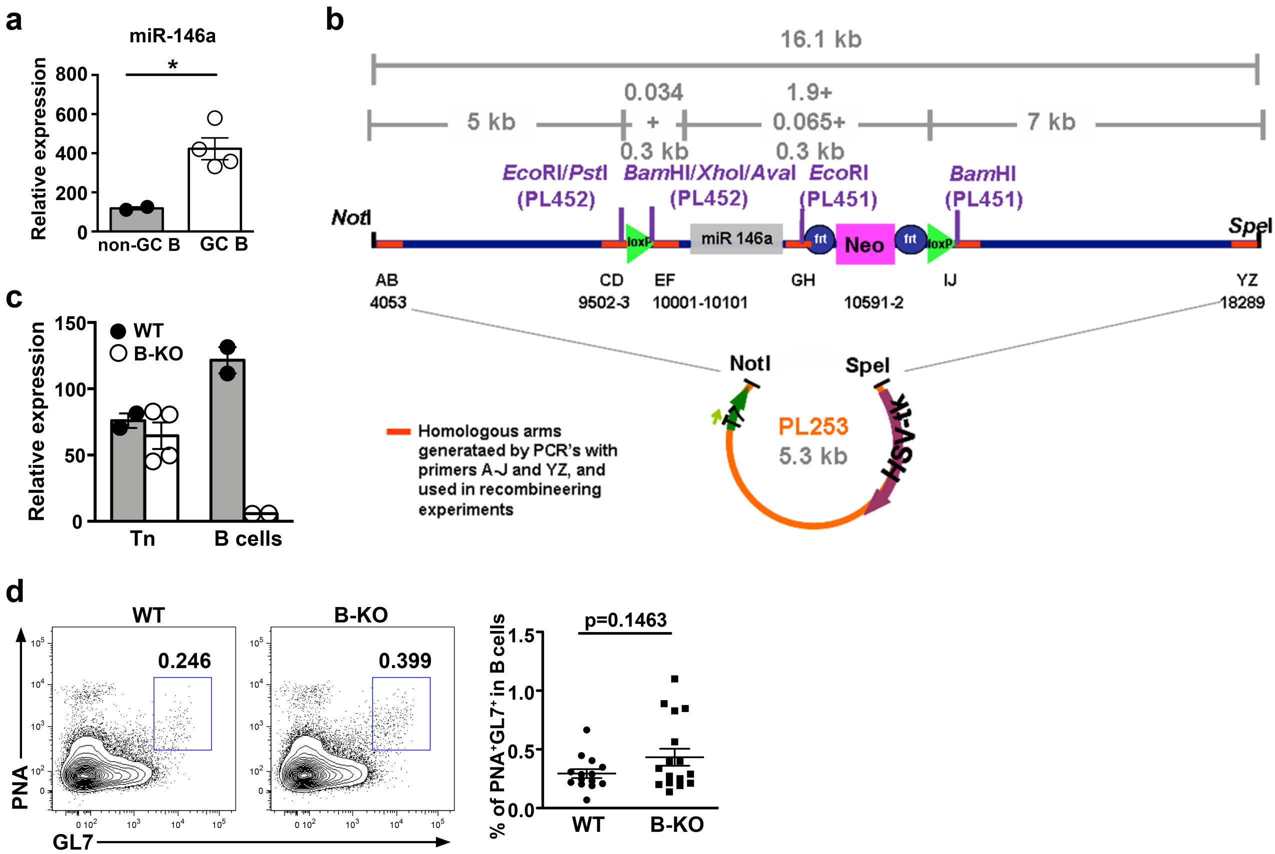

Supplementary Figure 1. B cell-specific miR-146a ablation does not lead to spontaneous accumulation of GC B cells at young ages. (a) Quantitative PCR of miR-146a levels in naïve mature B and GC B cells. **(b)** Schematic representation of the targeting strategy for *miR-146a^fl^* mice. **(c)** Expressions of miR-146a in naïve T cells (Tn) and B cells isolated from B-KO mice**. (d)** FACS analyses and frequencies of $PNA^{+}GL7^{+}$ GC B cells from spleens of ~8 wks old B-KO mice or their WT littermates. The data are shown as mean ± SD and are representative of three independent experiments (n=3-6). Each symbol represents an individual mouse, and the bar represents the mean. *p<0.05.

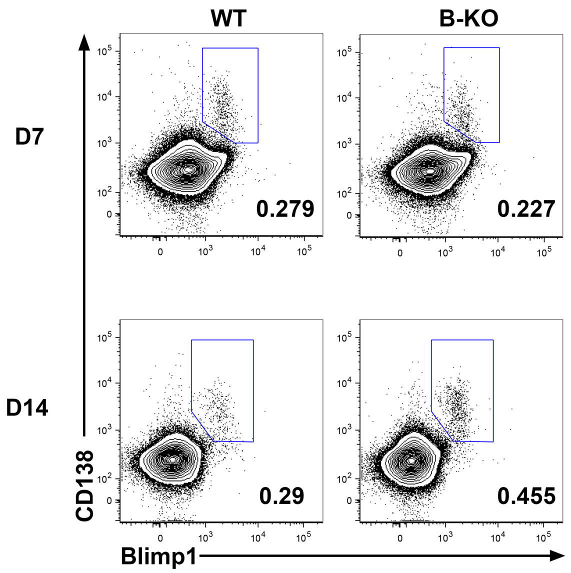

Supplementary Figure 2. Increased plasma cell production in mice with B cell-specific miR-146a ablation upon SRBC immunization. FACS analyses of $CD138^{+}Blimp1^{+}$ plasma cells from spleens of ~8 wks old B-KO mice or their WT littermates at indicated time points post SRBC immunization. The data are representative of two independent experiments (n=3-8).

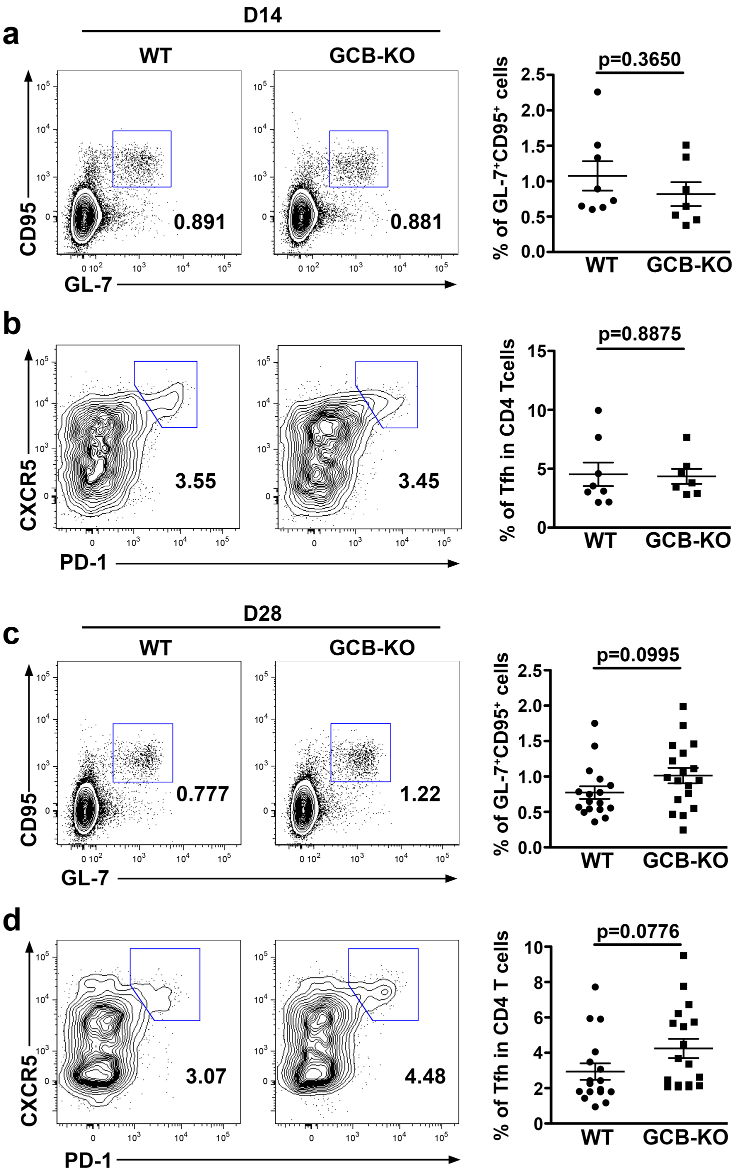

Supplementary Figure 3. GC B cell-specific miR-146a ablation has limited impact on GC responses. FACS analyses and frequencies of **(a, c)** $CD95^+GL7^+$ GC B cells and **(b, d)** $CXCR5^+PD\text{-}1^+$ Tfh cells in spleen from ~8 wks old *Cg1-cre miR-146a*$^{fl/fl}$ (GCB-KO) mice or their WT littermates at days 14 and 28 after SRBC immunization. Data are representative of 3 independent experiments. Each symbol represents an individual mouse, and the bar represents the mean.

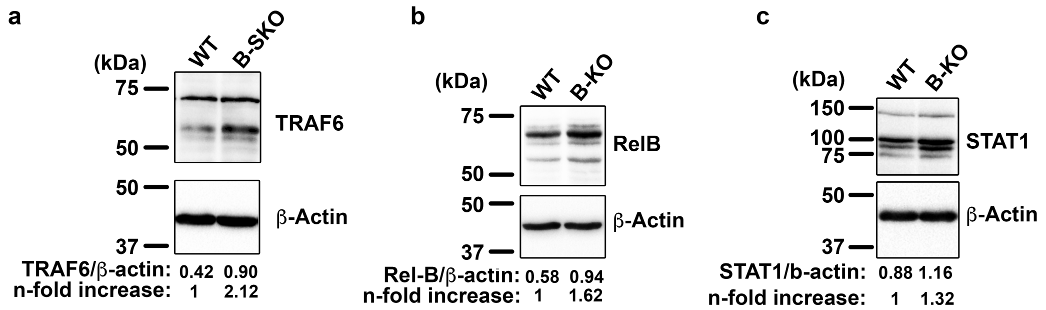

Supplementary Figure 4. Protein expressions of previously identified miR-146a targets in B cells. Immunoblot analysis of **(a)** TRAF6, **(b)** RelB and **(c)** STAT1 expression in B cells in the presence or absence of miR-146a. Densitometric expression values of each molecule were normalized to β-actin expression values and n-fold increase on the basis of each corresponding WT. Data are representative of three independent experiments (n=3-6).

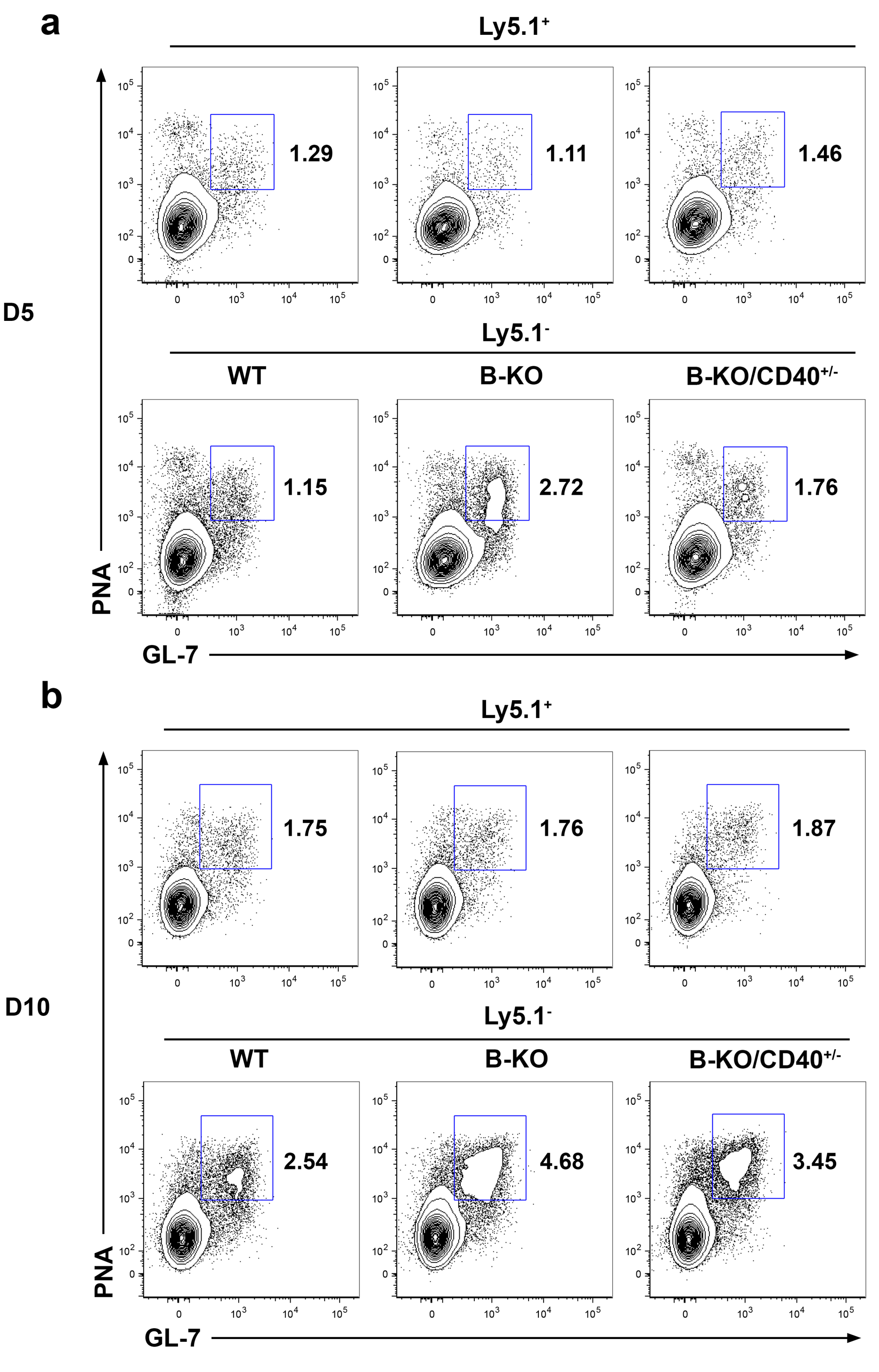

Supplementary Figure 5. miR-146a/CD40 axis plays a key role in regulating GC responses at both early and peak time points upon SRBC immunization. FACS analyses of Ly5.1^{-} and Ly5.1^{+} PNA^{+}GL7^{+} GC B cells in spleen from indicated mixed BM chimeras at days **(a)** 5 and **(b)** 10 after SRBC immunization. The data are representative of three independent experiments (n=5-10).

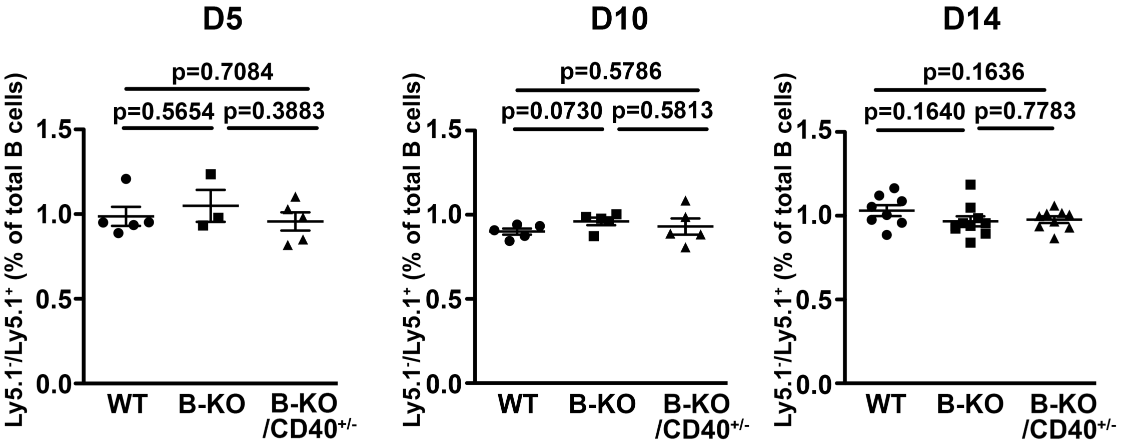

Supplementary Figure 6. miR-146a deficiency with or without CD40 heterozygosity did not impact total B cell frequencies in mixed BM chimeras. Ratios of frequencies of Ly5.1- and Ly5.1+ total B cells in spleen from indicated chimeric mice at days 5, 10 and 14 days after SRBC immunization. Data are representative of 3 independent experiments. Each symbol represents an individual mouse, and the bar represents the mean.

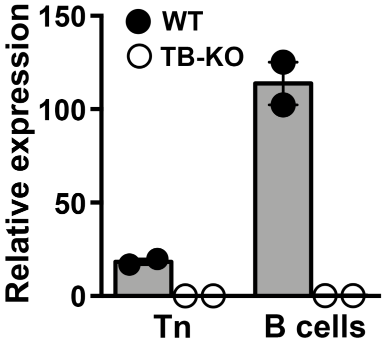

Supplementary Figure 7. Deletion of miR-146a in both T and B cells. Quantitative PCR of miR-146a levels in naïve T cells (Tn) and B cells isolated from TB-KO mice**.** The data are shown as mean ± SD and are representative of three independent experiments (n=3-6).

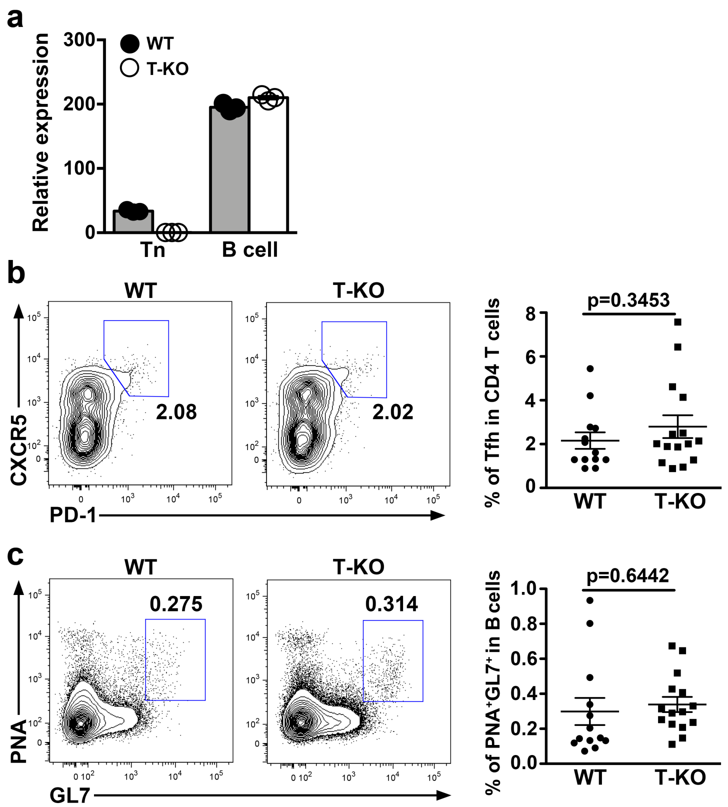

Supplementary Figure 8. Unaltered Tfh and GC B cell phenotypes in mice with T cell-specific miR-146a ablation at steady state. (a) Quantitative PCR of miR-146a levels in naïve T cells (Tn) and B cells isolated from T-KO mice**.** FACS analyses and frequencies of **(b)** CXCR5$^+$PD-1$^+$ Tfh cells or **(c)** PNA$^+$GL7$^+$ GC B cells from spleens of ~8 wks old T-KO mice or their WT littermates. The data are shown as mean ± SD and are representative of three independent experiments. Each symbol represents an individual mouse, and the bar represents the mean.

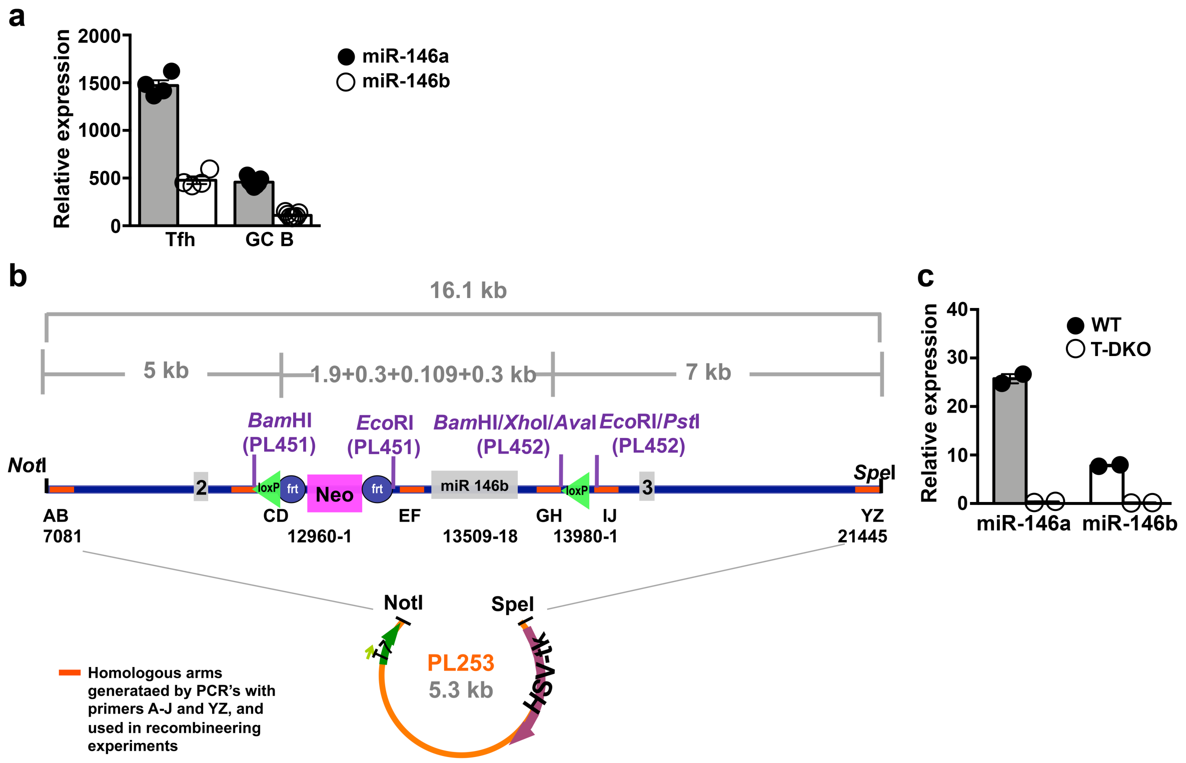

Supplementary Figure 9. Generation of mice with conditional alleles of miR-146b. (a) Quantitative PCR of miR-146a and miR-146b levels in Tfh and GC B cells at day 14 post SRBC immunization. **(b)** Schematic representation of the targeting strategy forb *miR-146b*^{fl} mice. **(c)** Expressions of miR-146a or miR-146b in naïve T cells (Tn) cells isolated from T-DKO mice**.** The data are shown as mean ± SD and are representative of three independent experiments (n=3-6).

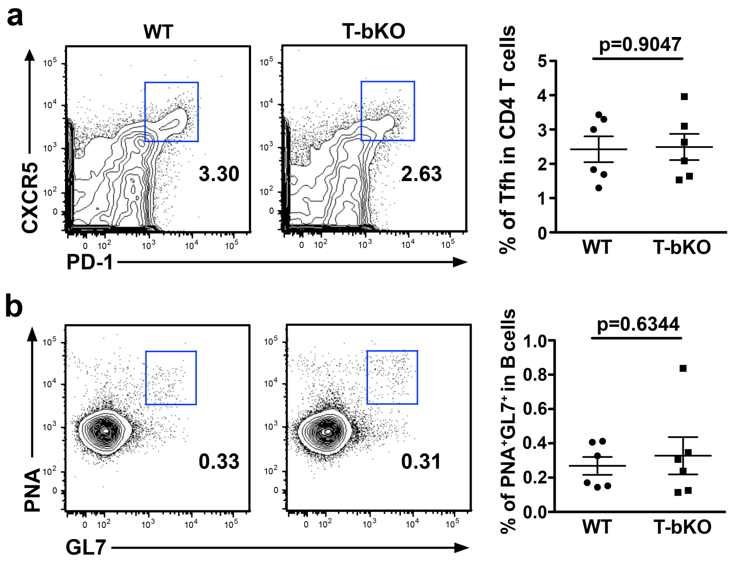

Supplementary Figure 10. Unaltered Tfh and GC B cell phenotypes in mice with T cell-specific miR-146b ablation (T-bKO) at steady state. FACS analyses and frequencies of **(a)** CXCR5^{+}PD-1^{+} Tfh cells or **(b)** PNA^{+}GL7^{+} GC B cells from spleens of ~8 wks old T-bKO mice or their WT littermates. The data are shown as mean ± SD and are representative of three independent experiments. Each symbol represents an individual mouse, and the bar represents the mean.

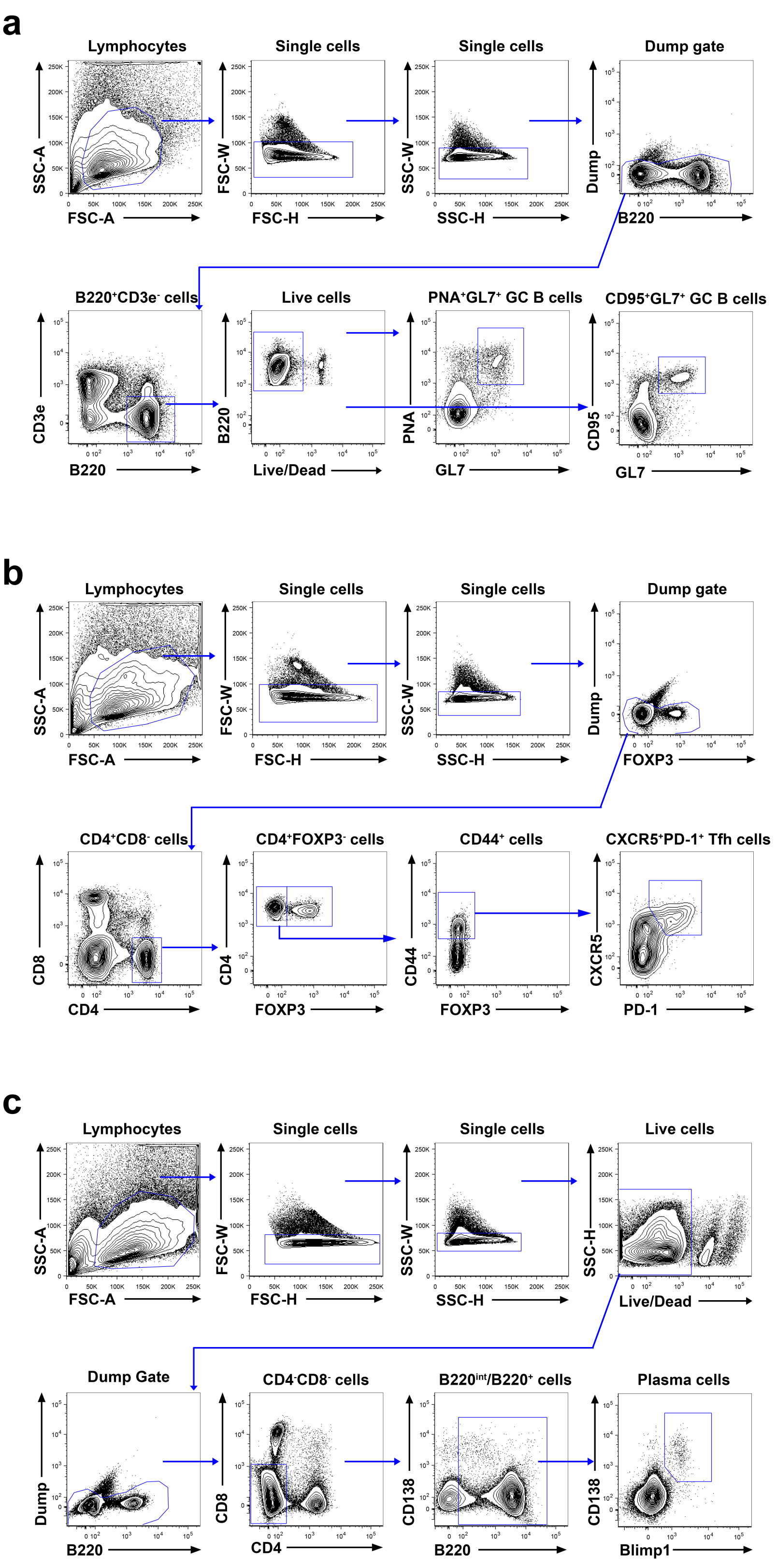

Supplementary Figure 11. Gating strategy of **(a)** PNA$^+$GL7$^+$ GC B cells and CD95$^+$GL7$^+$ GC B cells, **(b)** CXCR5$^+$PD-1$^+$ Tfh Cells and **(c)** CD138$^+$Blimp1$^+$ plasma cells in supplementary Figure 2.

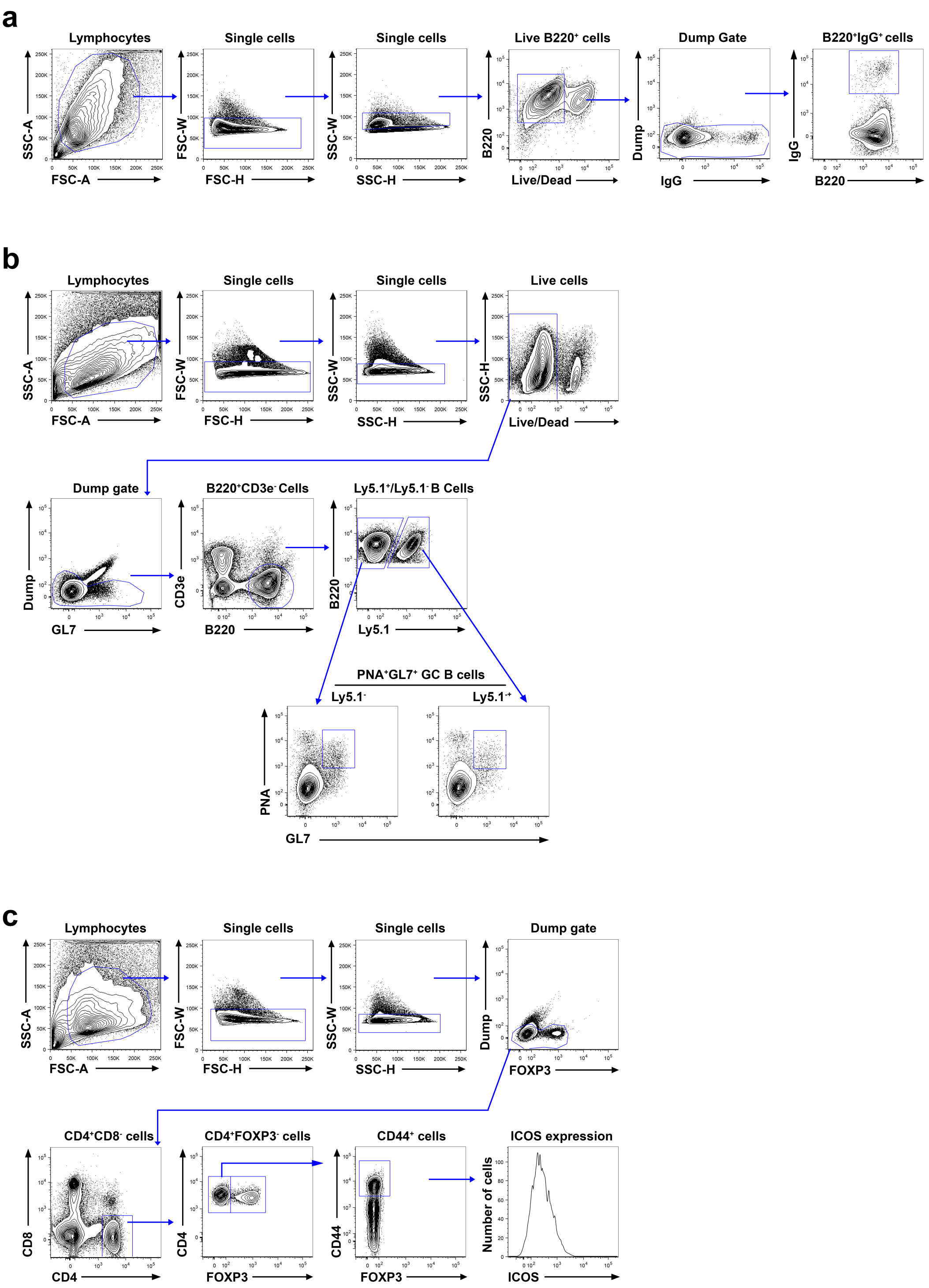

Supplementary Figure 12. Gating strategy of **(a)** IgG+ B cells in Figure 4c, **(b)** PNA+GL7+ GC B cells of mixed BM chimeras in Figure 4e and supplementary Figure 5 and **(c)** MFI of ICOS in Figure 7f.

Figure 3 d, h

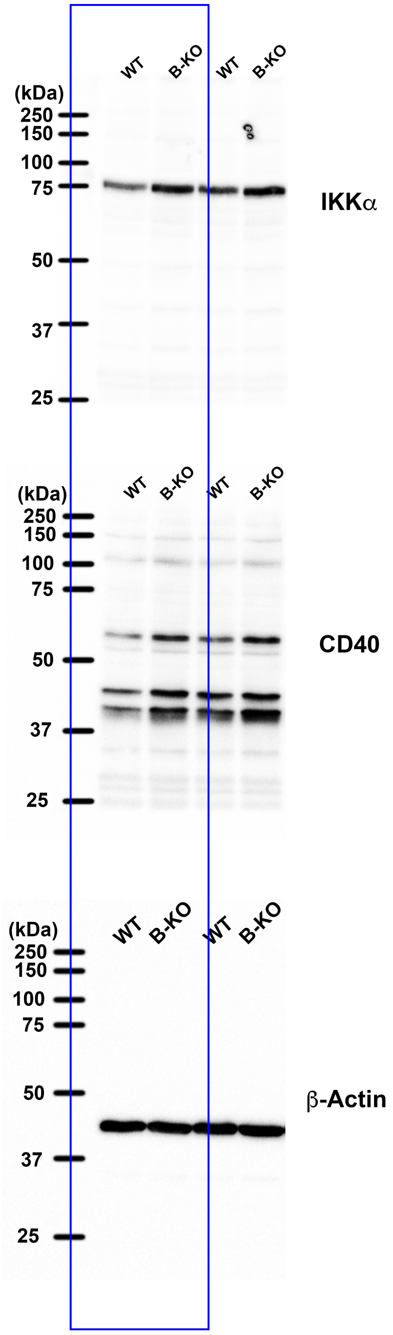

Figure 3 j, Supplementary Figure 4 a

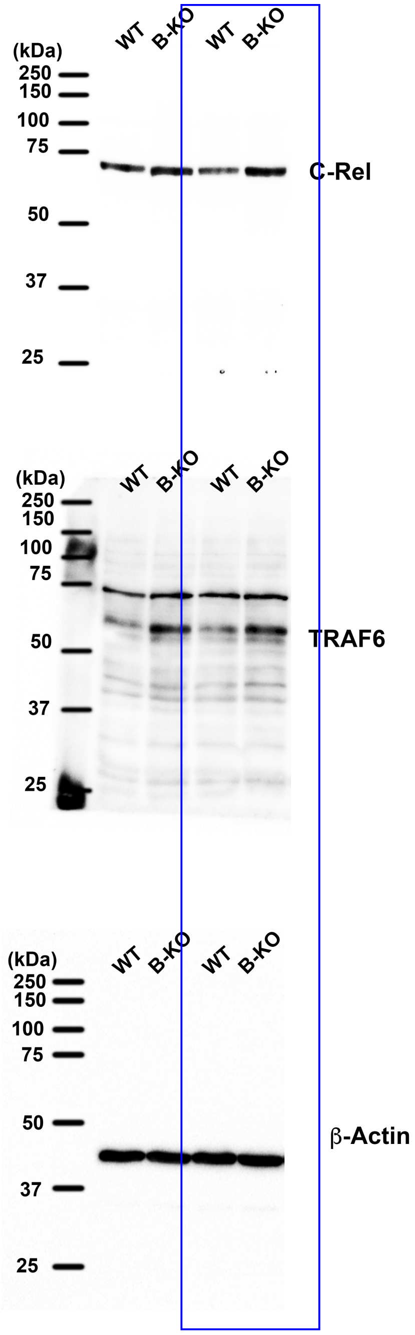

Supplementary Figure 4 b

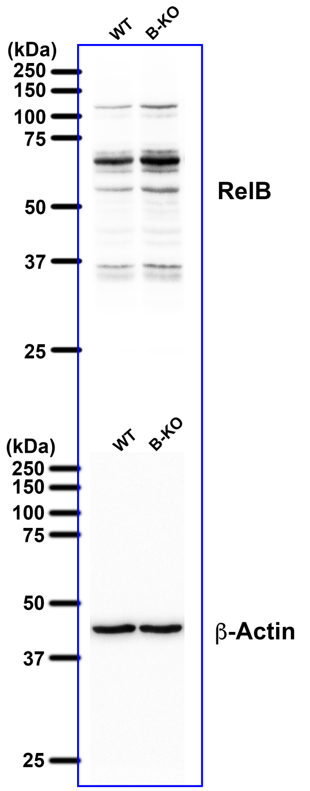

Supplementary Figure 4 c

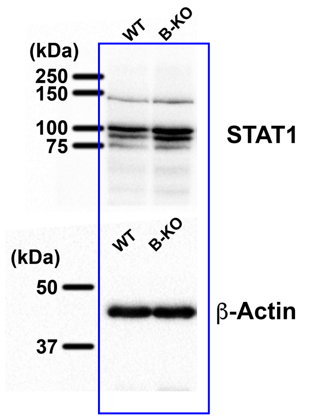

Supplementary Figure 13. Uncropped images of immunoblots

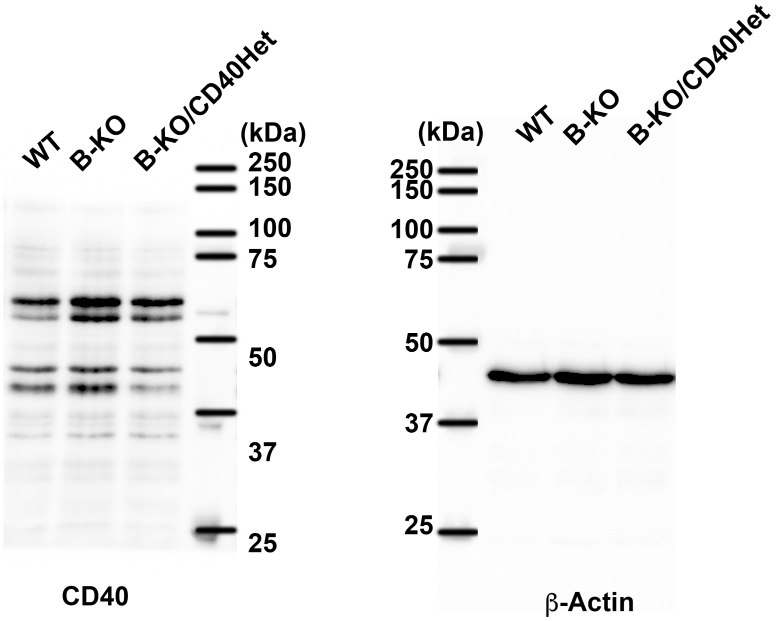

Supplementary Figure 14. Uncropped images of immunoblots in Figure 4a
