# Supplementary material for: Differential cell-intrinsic regulations of germinal center B and T cells by miR-146a and miR-146b
Source: Nat Commun. 2018 Jul 16;9:2757. doi: 10.1038/s41467-018-05196-3 (PMC6048122; doi:10.1038/s41467-018-05196-3)
Supplement: Supplementary file 3 — Description of Additional Supplementary Files [file 41467_2018_5196_MOESM3_ESM.pdf]

Description of Additional Supplementary Files

File Name: Supplementary Data 1
Description: A list of antibodies used in this manuscript.

File Name: Supplementary Data 2
Description: A list of reagents used in this manuscript.
